# Special report of the RSNA COVID-19 task force: systematic review of outcomes associated with COVID-19 neuroimaging findings in hospitalized patients

**DOI:** 10.1259/bjr.20210149

**Published:** 2021-04-29

**Authors:** Monique A. Mogensen, Pattana Wangaryattawanich, Jason Hartman, Christopher G. Filippi, Daniel S. Hippe, Nathan M. Cross, RSNA COVID-19 task force

**Affiliations:** 1 Department of Radiology, University of Washington School of Medicine, Seattle, WA, United States; 2 Department of Radiology, Tufts University School of Medicine, Boston, MA, United States

## Abstract

**Objective:**

We reviewed the literature to describe outcomes associated with abnormal neuroimaging findings among adult COVID-19 patients.

**Methods:**

We performed a systematic literature review using PubMed and Embase databases. We included all studies reporting abnormal neuroimaging findings among hospitalized patients with confirmed COVID-19 and outcomes. Data elements including patient demographics, neuroimaging findings, acuity of neurological symptoms and/or imaging findings relative to COVID-19 onset (acute, subacute, chronic), and patient outcomes were recorded and summarized.

**Results:**

After review of 775 unique articles, a total of 39 studies comprising 884 COVID-19 patients ≥ 18 years of age with abnormal neuroimaging findings and reported outcomes were included in our analysis. Ischemic stroke was the most common neuroimaging finding reported (49.3%, 436/884) among patients with mortality outcomes data. Patients with intracranial hemorrhage (ICH) had the highest all-cause mortality (49.7%, 71/143), followed by patients with imaging features consistent with leukoencephalopathy (38.5%, 5/13), and ischemic stroke (30%, 131/436). There was no mortality reported among COVID-19 patients with acute disseminated encephalomyelitis without necrosis (0%, 0/8) and leptomeningeal enhancement alone (0%, 0/12). Stroke was a common acute or subacute neuroimaging finding, while leukoencephalopathy was a common chronic finding.

**Conclusion:**

Among hospitalized COVID-19 patients with abnormal neuroimaging findings, those with ICH had the highest all-cause mortality; however, high mortality rates were also seen among COVID-19 patients with ischemic stroke in the acute/subacute period and leukoencephalopathy in the chronic period.

**Advances in knowledge:**

Specific abnormal neuroimaging findings may portend differential mortality outcomes, providing a potential prognostic marker for hospitalized COVID-19 patients.

## Introduction

Coronavirus disease 2019 (COVID-19), first reported in December 2019, has spread rapidly via person-to-person transmission leading to a global pandemic.^1^ As of January 4, 2021, there were over 83 million confirmed cases of COVID-19 and almost 2 million deaths reported to the World Health Organization.^2^ The most common manifestation of COVID-19 infection is respiratory symptoms, but the virus can cause complications in many organ systems including the neurological system.^1,3^ There is growing literature regarding a spectrum of imaging features associated with neurological complications in COVID-19 such as acute ischemia, intracranial hemorrhage (ICH), white matter signal abnormality, leptomeningeal enhancement, and hypoxic-ischemic injury owing to respiratory failure^3^; however, the association of abnormal neuroimaging findings with patient clinical outcomes remains largely undetermined.

Neurological complications in COVID-19 likely lead to greater morbidity and mortality.^4^ Since the onset of the pandemic, descriptive studies of abnormal neuroimaging findings among COVID-19 patients have been rapidly reported in the medical literature.^5^ Understanding the potential impact that abnormal neuroimaging findings have on the clinical course of COVID-19 patients is an important endeavor that can inform clinical management protocols. Our objective was to synthesize the available medical literature that describes specific abnormal neuroimaging findings and associated outcomes among patients hospitalized with confirmed COVID-19 diagnoses to lend insight into the management of COVID-19 patients with neurologic symptoms. Our primary patient outcome was all-cause in-hospital mortality, and our secondary patient outcomes included hospitalization status, discharge disposition, and the modified Rankin Scale (mRS) score at study end points.

## Methods and materials

### Search strategy

This systematic review followed the preferred reporting items for systematic reviews and meta-analysis (PRISMA) statement recommendations.^6^ A formal systematic review protocol was not registered in advance of performing the review. We performed an initial pilot search to determine relevant keywords and search terms. We identified a test cohort of relevant articles from manually searching that we compared against our pilot search results to ensure the adequacy of our final comprehensive search. On October 31, 2020, we searched two databases (PubMed via PubMed.gov and Embase via Embase.com) utilizing both medical subject headings (MeSH) and Embase subject headings (Emtree terms) combined with keywords related to COVID, neurologic disorders and journals, and diagnostic imaging and journals published between the dates of October 1, 2019 and October 31, 2020. The full search strategy can be found in Supplementary Material 1.

Supplementary Material 1.

### Study selection criteria and process

Two independent reviewers (MAM and PW) screened article titles and abstracts obtained through the PubMed and Embase searches using the web-based software Covidence (Covidence Systematic Review Software, Veritas Health Innovation, Melbourne, Australia) to identify original articles reporting on the association between neuroimaging findings (CT, CT angiography (CTA), MRI, MR angiography (MRA) only) in hospitalized patients with COVID-19 and their outcomes.^7^ Inclusion criteria included COVID-19 positive patients (all or majority of subjects with confirmed positive COVID-19 reverse transcription polymerase chain reaction (RT-PCR) swab or immunoglobulin (Ig) serology) with relevant or positive neuroimaging results and outcomes data reported. Exclusion criteria included editorials or letters to the editor, review articles, patients <18 years old, outcomes data not relatable to specific neuroimaging findings, case reports or case series with <5 patients, studies performed in non-hospital settings, and absence of availability of an English translation. Titles and abstracts were classified as “Yes”, “No”, or “Maybe.” In cases of disagreement or “Maybe”, a third reviewer (NMC) performed arbitration with the two original reviewers. Two reviewers (MAM and PW) then performed full text reviews of articles classified as “Yes” utilizing the same criteria as used for screening. Full text articles were placed into “Include” or “Exclude” categories with exclusion reasons given. Conflicts were resolved by a consensus among a combination of the three reviewers (MAM, PW, NMC).

The quality of included studies was examined based on the National Institutes of Health (NIH) Quality Assessment Tool for Case Series Studies (9 items) and Observation Cohort and Cross-Sectional Studies (14 items) by two reviewers (JH and PW).^8^ Studies with an overall rating of “Good” or “Fair” were included in our systematic review. Since all articles are publicly available, no institutional review board approval was required for this study.

### Data extraction and synthesis

Study titles, authors, study design, dates of data collection, country of origin, patient demographics (age, sex, and sample size), main neurological imaging findings (imaging modalities, descriptions of findings, and/or interpretations), neurological symptoms, acuity of neuroimaging findings or neurological symptoms prompting imaging relative to COVID-19 symptom onset or diagnosis, and patient outcomes (all-cause in-hospital mortality, hospitalization status, discharge disposition, and/or mRS score) were systematically recorded from the included studies by one investigator (MAM) and audited by a second investigator (PW). Patients with normal or incidental imaging findings only were not included in our analysis. Microsoft Excel (Microsoft, Redmond, WA) was used for data categorization and descriptive statistics and results were summarized in evidence tables. Due to heterogeneity among study populations and outcomes data reported, a meta-analysis was not performed.

## Results

### Study selection, design and quality assessment

**Figure 1.**
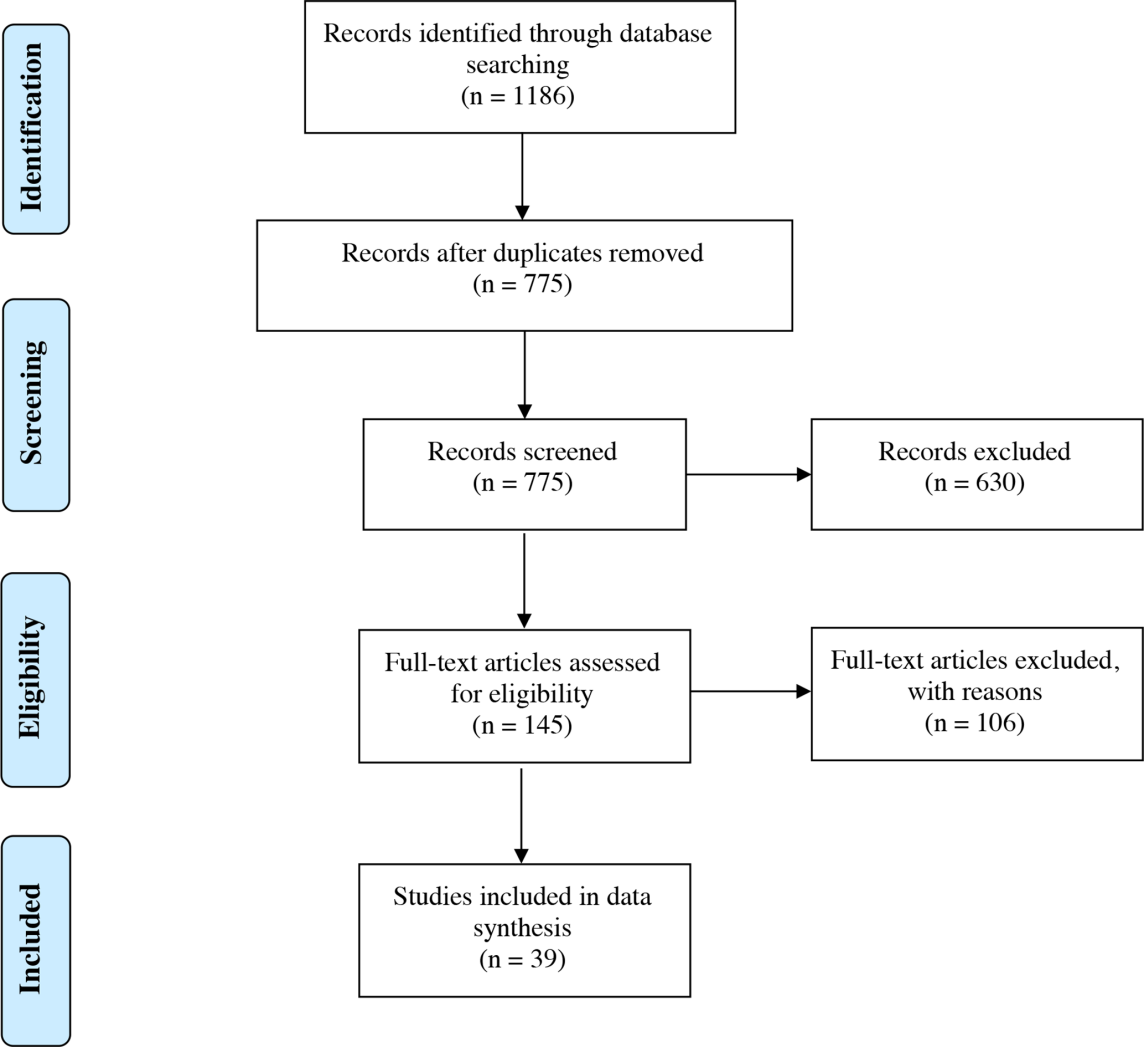
Preferred Reporting Items for Systematic Reviews and Meta-Analyses (‘PRISMA’) flow chart.

Initially, 1186 articles were identified through the PubMed and Embase searches and 411 duplicates were removed leaving 775 unique articles for title and abstract screening (Figure 1) . Of 775 unique articles, 630 were excluded at title and abstract screening, leaving 145 studies for full-text review. Of these 145 studies, 106 were excluded at full-text review for various reasons (see Supplementary Material 2) leaving 39 studies for quality assessment and data extraction. The methodological quality of all 39 studies was rated as “Good” or “Fair” by the NIH quality assessment tools (see Supplementary Material 3). Of these 39 studies included in our review, the majority were based on data from the United States (20),^9–28^ followed by France (4),^29–32^ Italy (3),^33–35^ the United Kingdom (2),^36,37^ Spain (2),^38,39^ China (1),^40^ Turkey (1),^41^ United Arab Emirates (1),^42^ Iran (1),^43^ Switzerland (1),^44^ India (1),^45^ and Germany (1).^46^ One study contained combined data from 16 different countries.^4^ Dates reported for clinical data collection ranged from as early as January 8, 2020 to as late as August 31, 2020. All study designs were either retrospective case series or retrospective cohort studies. A total of 28 articles reported data from a single institution, while 11 reported data from multiple institutions.

Supplementary Material 2.

Supplementary Material 3.

### Patient population and neuroimaging features

Overall, we collected data on a total of 884 COVID-19 patients with reported abnormal neuroimaging findings and associated outcomes data. A total of 18 studies reported individual patient ages in years and 15 other studies reported mean ages in years. Combined, the mean age across studies that provided these data was 61.7 years (*n* = 554 patients). Sex was reported for 813 of the patients and the majority were male (66.7%, 542/813). Of the 884 patients, 28 did not have specific information on the neurological symptoms that prompted imaging. In 96.2% of patients (850/884), neurological symptoms or National Institutes of Health Stroke Scale (NIHSS) scores were reported and associated with obtaining neurological imaging. In six patients, the neuroimaging findings were discovered incidentally. Neuroimaging findings, regardless of imaging modality (CT, MR, CTA, MRA) were separated into multiple categories (Table 1) including: (1) all-cause stroke (82.5%, 729/884) with subcategories of ischemic stroke (49.3%, 436/884), non-specified stroke (17.0%, 150/884) and ICH (16.2%, 143/884) when reported separately, (2) encephalitis (3.8%, 34/884) with subcategories of non-specific encephalitis (1.2%, 11/884), limbic encephalitis (2.1%, 19/884), and acute necrotizing encephalitis (ANE) (0.5%, 4/884), (3) leukoencephalopathy alone (1.5%, 13/884), (4) microhemorrhages alone (0.8%, 7/884), (5) combined leukoencephalopathy, microhemorrhages, and/or all-cause stroke (7.7%, 68/884), (6) leptomeningeal enhancement (1.4%, 12/884), (7) acute disseminated encephalomyelitis (ADEM) (1.0%, 9/884) with or without microhemorrhage or necrosis, and (8) other (1.4%, 12/884) for less common neuroimaging features with four or less patients with outcomes reported. The category of combined leukoencephalopathy, microhemorrhages, and/or all-cause stroke captures data from papers that reported imaging findings as leukoencephalopathy and/or microhemorrhage, or patients with leukoencephalopathy who also had a combination of microhemorrhage, ICH, and/or ischemic stroke. Neurological symptoms prompting imaging or neuroimaging findings were classified as acute (≤7 days), subacute (8–21 days), or chronic (≥22 days) relative to COVID-19 related symptom onset or diagnosis when reported either at the patient level or as a mean or median (Table 1). Among patients with reported time since COVID onset, the majority of neurological events prompting imaging or neuroimaging findings were subacute or chronic in nature, except for in ischemic stroke where neuroimaging findings were typically acute or subacute. However, the temporal relationship between COVID onset and neuroimaging was not reported for 33.7% (298/884) of patients.

**Table 1.** Neuroimaging findings in COVID-19 patients and time since COVID-19 symptom onset or diagnosis

Neuroimaging finding (*n* = row totals) total *n* = 884	Time since onset of COVID-19 symptoms or diagnosis (n of patients reported on)^*a*^			
	**Acute**	**Subacute**	**Chronic**	**Not Reported**
**Stroke (729**)				
Non-specified stroke (150)	--	57	--	93
Ischemic stroke (436)	212	85	7	132
ICH (143)	5	65	21	52
**Encephalitis (34**)				
Non-specific encephalitis (11)	7	3	--	1
Limbic encephalitis (19)	--	19	--	0
Acute necrotizing encephalitis (4)	--	2	1	1
**Leukoencephalopathy alone** (13)	--	2	8	3
**Microhemorrhages alone** (7)	4	--	2	1
**Combined leukoencephalopathy ± microhemorrhages**±**all-cause stroke** (68)	1	1	60	6
**Leptomeningeal enhancement alone** (12)	1	11	--	0
**ADEM (9**)				
ADEM (4)	--	3	1	0
ADEM with microhemorrhage (4)	1	0	2	1
ADEM with microhemorrhage and necrosis (1)	--	1	--	--
**Other** (12)				
Hypoxic-ischemic injury ± microhemorrhages (4)	--	1	--	3
PRES ± hemorrhages (2)	--	--	--	2
Acute tumefactive demyelination (1)	--	--	--	1
Transient CLOCC + SAH (1)	--	--	--	1
Pseudotumor cerebri (1)	--	1	--	0
Status epilepticus with cortical changes (1)	--	--	1	0
Post-infectious myelitis (1)	--	1	--	0
Communicating hydrocephalus (1)	--	--	--	1

ADEM, acute disseminated encephalomyelitis; CLOCC, cytotoxic lesions of the corpus callosum; ICH, intracranial hemorrhage; PRES, posterior reversible encephalopathy syndrome; SAH, subarachnoid hemorrhage.

a Time since onset of COVID-19: acute (≤7 days), subacute (8–21 days), or chronic (≥22 days).

### Neuroimaging features and patient outcomes

Data on reported hospital outcomes were collected from each study (Table 2). There was high variation across study population characteristics, documented imaging features, and clinical outcomes reported at the end of each study period, limiting data pooling and preventing meta-analysis. Nevertheless, our main outcome of all-cause mortality during hospitalization was reported for all patients in all articles up to the study end points. Overall, among the major imaging finding groups with reported mortality outcomes, the highest all-cause mortality was observed among patients with leukoencephalopathy (38.5%, 5/13), followed by all-cause stroke (33.2% 242/729), and then combined leukoencephalopathy, microhemorrhages, and/or stroke (20.6%, 14/68). Among patients with stroke neuroimaging features, those in the subgroup with ICH had the highest all-cause mortality rate (49.7%, 71/143) across all study groups or subgroups. Patients with only microhemorrhages and encephalitis as imaging findings had lower all-cause mortality rates (14.3% (1/7) and 11.8% (4/34), respectively), although encephalitis mortality was higher when the limbic encephalitis and ANE subgroups were excluded (27.3%, 3/11) and when encephalitis patients had an acute presentation (75%, 3/4). Patients with either leptomeningeal enhancement alone or ADEM without necrosis fared the best with 0% reported mortality for both groups (0/12 and 0/8, respectively).

**Table 2.** Neuroimaging findings in COVID-19 patients and reported outcomes at study end points

Neuroimaging finding	All-cause mortality during hospitalization % (n/total)^*a*^	Remained hospitalized total n, if reported	Discharged total n, if reported	Survivor mRS score at study endpoints
**Stroke**	**33.2%** (**242/729**)			
Ischemic stroke	30.0% (131/436)	26	175	Mean 3.36 (*n* = 168)
ICH	49.7% (71/143)	12	28	Range 4–5 (*n* = 11)^*b*^; 3 (*n* = 1)
Non-specified stroke	26.7% (40/150)	0	110	Mean 3.58 (*n* = 49)
**Encephalitis**	**11.8%** (**4/34**)			
Limbic encephalitis	5.3% (1/19)	NR	NR	Mean 1 (*n* = 2)
Acute necrotizing encephalitis	0.0% (0/4)	2	2	Mean 5 (*n* = 2)
Non-specific encephalitis	27.3% (3/11)	3	1	Mean 3.67 (*n* = 3)
**Leukoencephalopathy alone**	**38.5%** (**5/13**)	8	0	NR
**Microhemorrhages alone**	**14.3%** (**1/7**)	3	3	NR
**Combined leukoencephalopathy ± microhemorrhages±all-cause stroke**	**20.6%** (**14/68**)	4	6	Mean 4.87 (*n* = 39)
**Leptomeningeal enhancement alone**	**0%** (**0/12**)	NR	NR	1 (*n* = 1)
**ADEM**	**11.1%** (**1/9**)			
ADEM ± microhemorrhage	0.0% (0/8)	7	NR	2 (*n* = 1)
ADEM with necrosis + microhemorrhage	100.0% (1/1)	0	0	--
**Other**				
Hypoxic-ischemic injury ± Microhemorrhages	50.0% (2/4)	NR	1	NR
PRES ± hemorrhages	50.0% (1/2)	1	0	NR
Acute tumefactive demyelination	0% (0/1)	1	0	NR
Transient CLOCC + SAH	100% (1/1)	0	0	--
Pseudotumor cerebri	0% (0/1)	0	1	NR
Status epilepticus with cortical changes	0% (0/1)	1	0	NR
Post-infectious myelitis	0% (0/1)	0	1	NR
Communicating hydrocephalus	0% (0/1)	0	1	NR

ADEM, acute disseminated encephalomyelitis; CLOCC, cytotoxic lesions of the corpus collosum; ICH, intracranial hemorrhage; NR, not reported; PRES, posterior reversible encephalopathy syndrome; SAH, subarachnoid hemorrhage; mRS, modified Rankin Scale.

a Percent only provided for main outcome (all-cause mortality) given variable reporting of all other outcomes.

b One study only reported mRS scores as a range so mean could not be calculated.

Secondary outcomes, including the number of patients that remained hospitalized, the number discharged, and mRS scores at study endpoints were variably reported and incomplete for the majority of included studies. When reported, some patients categorized into each major neuroimaging finding category remained hospitalized (total reported, *n* = 68) or were discharged (total reported *n* = 329) at the end of reported study periods (Table 2). The mRS scores were provided for patients who survived in 11 studies. The reported mRS scores at the end of study periods suggested moderate-to-severe disability (mRS score ranging from 3 to 5) for patients with imaging findings consistent with ICH (mRS range 3–5, *n* = 12), ischemic stroke (mean mRS 3.36, *n* = 168), non-specified stroke (mean mRS 3.58, n=49), combined leukoencephalopathy, microhemorrhages, and/or ICH (mean mRS 4.87, *n* = 39), and non-limbic encephalitis (mean mRS 4.2, *n* = 5).

## Discussion

In this systematic review, we summarize patient outcomes associated with a range of potential COVID-19-associated neuroimaging features reported in the medical literature. A few prior systematic reviews have reported on specific neuroimaging findings among COVID-19 adult patients or on common neuroimaging findings associated with mild *vs* severe COVID-19 disease.^47,48^ Tan et al summarized outcomes for acute ischemic stroke in COVID-19 patients and found a high mortality rate (38%).^49^ Our study provides a more comprehensive review of all reported abnormal imaging findings among hospitalized COVID-19 patients regardless of acuity.

We found that across all major imaging feature subgroups, patients with ICH had the highest all-cause mortality (49.7%) followed by patients with imaging features consistent with leukoencephalopathy (38.5%) and ischemic stroke (30%). Notable, but based on limited data, no patients with leptomeningeal enhancement alone or ADEM without necrosis had died at the end of reported study periods; however, a majority of patients with ADEM remained hospitalized at last reported follow-up. Patients with encephalitis described as limbic or autoimmune encephalitis or ANE with a subacute presentation had lower mortality rates compared to patients with acute encephalitis demonstrating white matter and cortical imaging findings attributed to encephalitis. As expected, the temporal relationship between the development of COVID-19 symptoms and the onset of neurologic symptoms and/or neuroimaging findings tended to be acute to subacute for ischemic stroke and a subset of non-limbic encephalitis cases, and subacute to chronic for all other imaging findings. Leukoencephalopathy alone or leukoencephalopathy combined with microhemorrhage or ICH were predominantly chronic imaging findings.

Only a handful of the larger retrospective studies had any comparison group (*e.g.* non-COVID patients) or reported odds ratios (OR) for neuroimaging findings and mortality. In univariate analysis, Kvernland et al found that COVID-19 patients with ICH had a higher in-hospital mortality than patients with ICH without COVID-19 (84.6% *vs* 4.6%, *p* < 0.001).^17^ Melmed et al reported that the odds of mortality were higher for COVID-19 patients who had ICH (OR = 2.6, CI 1.2–5.9), *p* = 0.02) than COVID-19 patients without ICH.^21^ While we could not separate our analysis of ICH into different subtypes due to the lack of more granular data, one large retrospective cohort study suggested that mortality is higher among patients with multicompartmental ICH versus subdural, subarachnoid, or intraparenchymal hemorrhage alone.^12^ In addition, they found that higher mortality was associated with ICH in COVID-19 patients requiring mechanical ventilation (OR 10.24 [95% CI, 0.43–243], *p* = 0.015), with INR >1.2 (OR 14.36 [95% CI, 1.69–122], *p* = 0.015) and among patients presenting with spontaneous *vs* traumatic hemorrhages (OR 6.11 [95% CI, 0.31–119], *p* = 0.023).

Ischemic stroke was the most common neuroimaging finding reported in our review (49.3% of patients). Altschul et al found that patients with COVID-19 and emergent large vessel occlusion had a higher risk of mortality during the pandemic *vs* patients without COVID-19 (OR 16.63 [95% CI, 2.47–112], *p* = 0.004) and mortality was higher if patients were older than 60 years of age and had pulmonary symptoms.^11^ In-hospital mortality was also found to be higher in patients with large vessel occlusion who had COVID-19 *vs* those without COVID-19 (41.7% *vs* 11.8%, *p* = 0.025) by Escalard et al.^29^ Jain et al found higher mortality risk (OR = 6.02 [95% CI, 2.6–14.6], *p* < 0.001) in COVID-19 patients with neuroimaging findings, the vast majority of which were ischemic or hemorrhage stroke (92.5%).^15^ Ntaios et al was the largest study (174 patients) to report on mortality in large vessel acute ischemic stroke from 16 countries and found patients with COVID-19 had a higher risk of death (OR = 4.3 [95% CI, 2.22–8.30]) compared to matched COVID-19 negative patients.^4^

Even among COVID-19 survivors with abnormal neuroimaging findings, disability as measured by the mRS when reported was moderate-to-severe at study end points. This was true for COVID-19 patients with ICH, ischemic stroke, combined leukoencephalopathy, microhemorrhage, and/or ICH, and non-limbic encephalitis. In one study, COVID-19 survivors of all-cause stroke had moderate-to-severe disability scores (mean mRS 3.58 [SD 1.23], *n* = 49) that were significantly higher compared to COVID-19 negative controls (mRS 1.86 [SD 1.52], *p* < 0.001).^45^ One larger study also reported a median mRS of 4 (IQR, 2–6) for 125 patients with ischemic stroke compared to a median mRS of 2 (IQR, 1–4) for matched non-COVID ischemic stroke patients.^4^ These findings collectively suggest that COVID-19 patients, particularly stroke patients have considerable morbidity even if they survive.

This systematic review has multiple limitations. The absence of a formal, prospectively registered protocol increases the risk for potential bias during the review process. Much of our data is gathered from case series and small retrospective observational cohorts with significant heterogeneity in variables reported, limiting the ability to generalize neuroimaging related mortality data to all COVID-19 patients. Furthermore, our primary outcome data were limited to all-cause mortality as mortality specifically related to unique neuroimaging findings were not routinely reported. In some studies, neurological symptom onset prompting imaging was used in lieu of actual time of imaging to estimate chronicity of neuroimaging findings relative to COVID-19 symptom onset or diagnosis, which may underestimate chronicity in some cases. Confounding variables such as disease severity, co-morbidities, medications, and treatment were variably reported and thus not summarized in our review. Neuroimaging categories used such as encephalitis, ADEM, PRES, leukoencephalopathy, and hypoxic-ischemic injury were based on classification by study authors who often incorporated clinical data for diagnosis. Thus, there may be some overlap of specific imaging findings within categories and some uncertainty of appropriate categorization due to clinical bias. Some imaging finding groups had small sample sizes limiting conclusions that can be drawn from the data. Reporting standards also varied regarding what imaging modalities were associated with specific imaging findings, as many patients had multiple imaging exams and modalities recorded. Patients were not always followed until discharge or death and our review may underestimate mortality in groups where patients remained hospitalized such as ADEM and leukoencephalopathy. The pandemic has also changed the delivery of health care in many hospitals due to necessity, increased patient volumes, resource limitations, or limiting of non-emergent healthcare. The impact of these pandemic-related effects on patients being managed with COVID-19 is likely complex and may have an impact on outcomes as well. Finally, control data from patients with neurological symptoms with normal or incidental imaging findings were not included in this review to compare mortality rates, as our objective was to examine abnormal imaging findings and associated patient outcomes.

## Conclusions

We report the mortality rates associated with the most common neuroimaging findings reported among hospitalized COVID-19 patients during the first three quarters of the pandemic. In our comprehensive systematic review, patients with ICH had the highest mortality rate (49.7%) across all subgroups, followed by patients with leukoencephalopathy (38.5%) and ischemic stroke (30%). In studies reporting mortality data, ischemic stroke and ICH made up 82.5% of abnormal neuroimaging findings and ischemic stroke was the most common abnormal neuroimaging finding reported in COVID-19 patients (49.3%). Future studies reporting on neuroimaging features among COVID-19 patients should provide more detailed data on the full spectrum of imaging features, not only on stroke and ICH. Moreover, future studies can help fill a critical knowledge gap by reporting on a variety of outcomes directly associated with abnormal neuroimaging features including long-term outcomes for patients who remained hospitalized or were discharged to rehabilitation. Finally, inclusion of a control population of patients with COVID-19 with normal neuroimaging findings during the study period would be helpful to inform clinical teams on the incidence and expected clinical courses for hospitalized COVID-19 patients with specific abnormal neuroimaging features.
