# Supplementary material for: Special report of the RSNA COVID-19 task force: systematic review of outcomes associated with COVID-19 neuroimaging findings in hospitalized patients
Source: Br J Radiol. 2021 Apr 29;94(1127):20210149. doi: 10.1259/bjr.20210149 (PMC8553187; doi:10.1259/bjr.20210149)
Supplement: Supplementary Material 1. [file bjr.20210149.suppl-01.docx]

**Supplementary Material: Search Strategy**

| **Databases** | **PubMed, Embase** |
| --- | --- |
| **Date Searched** | **10/31/2020** |
| **Total Results** | **1186** |
| **Duplicates Removed** | **411** |
| **Unique Results** | **775** |
| **Concepts** | 1. **COVID** 2. **Neurologic Disorder** 3. **Diagnostic Imaging (MRI/CT)** |

PubMed

("COVID"[tiab] OR "COVID"[ot] OR "COVID-19"[tiab] OR "COVID-19"[ot] OR "COVID-2019"[tiab] OR "COVID-2019"[ot] OR "COVID19"[tiab] OR "COVID19"[ot] OR "SARS-CoV-2"[tiab] OR "SARS-CoV-2"[ot] OR "Coronavirus"[tiab] OR "Coronavirus"[ot] OR "2019-nCoV"[tiab] OR "2019-nCoV"[ot] OR "nCoV-2019"[tiab] OR "nCoV-2019"[ot] OR "severe acute respiratory syndrome coronavirus 2"[nm] OR "COVID-19"[nm] OR )

AND

("neurologic"[tiab] OR "neurologic"[ot] OR "CNS"[tiab] OR "CNS"[ot] OR "nervous system"[tiab] OR "nervous system"[ot] OR "brain"[tiab] OR "brain"[ot] OR "cerebr*"[tiab] OR "cerebr*"[ot] OR "neurovascular"[tiab] OR "neurovascular"[ot] OR "cerebrovascular"[tiab] OR "cerebrovascular"[ot] OR "stroke"[tiab] OR "stroke"[ot] OR "cerebral vein thrombosis"[tiab] OR "cerebral vein thrombosis"[ot] OR "neuropath*"[tiab] OR "neuropath*"[ot] OR "*encephalit*"[tiab] OR "*encephalit*"[ot] OR "meningit*"[tiab] OR "meningit*"[ot] OR "leukoencephalopath*"[tiab] OR "leukoencephalopath*"[ot] OR "Nervous System Diseases"[Mesh] OR "Central Nervous System Diseases"[Mesh] OR "Cranial Nerve Diseases"[Mesh] OR "Demyelinating Diseases"[Mesh] OR "Stroke"[Mesh] OR "Brain"[Mesh] OR "Nervous System"[Mesh] OR "Intracranial Hemorrhages"[Mesh] OR "Hematoma, Subdural"[Mesh] OR "pituitary"[tiab] OR "pituitary"[ot] OR "intracran*"[tiab] OR "intracran*"[ot] OR "intracereb*"[tiab] OR "intracereb*"[ot] OR "hemispher*"[tiab] OR "hemispher*"[ot] OR "vertebrobas*"[tiab] OR "vertebrobas*"[ot] OR "infratentorial"[tiab] OR "infratentorial"[ot] OR "supratentorial"[tiab] OR "supratentorial"[ot] OR "anterior circulat*"[tiab] OR "anterior circulat*"[ot] OR "posterior circulat*"[tiab] OR "posterior circulat*"[ot] OR "infarct*"[tiab] OR "infarct*"[ot] OR "cerebell*"[tiab] OR "cerebell*"[ot] OR "cognit*"[tiab] OR "cognit*"[ot] OR "neuro*"[tiab] OR "neuro*"[ot] OR "JAMA Neurology"[Journal] OR "Journal of Neurology"[Journal] OR "Neurology"[Journal] OR "Journal of neurosurgery"[Journal] OR "Annals of neurology"[Journal] OR "Journal of neurointerventional surgery"[Journal] OR "Neurosurgery"[Journal] OR "The New England journal of medicine"[Journal] OR "Stroke"[Journal] OR "The Lancet. Neurology"[Journal] OR "Acta neuropathologica"[Journal] OR "Spine"[Journal] OR "European Journal of Neurology"[Journal] OR )

AND

("Neuroimaging"[tiab] OR "Neuroimaging"[ot] OR "Magnetic resonance imaging"[tiab] OR "Magnetic resonance imaging"[ot] OR "MRI"[tiab] OR "MRI"[ot] OR "computed tomograph*"[tiab] OR "computed tomograph*"[ot] OR "CT"[tiab] OR "CT"[ot] OR "Imaging"[tiab] OR "Imaging"[ot] OR "CAT scan*"[tiab] OR "CAT scan*"[ot] OR "MR Imaging"[tiab] OR "MR Imaging"[ot] OR "fMRI"[tiab] OR "fMRI"[ot] OR "Magnetic Resonance Imaging"[Mesh:NoExp] OR "Tomography, X-Ray Computed"[Mesh:NoExp] OR "Tomography"[Mesh:NoExp] OR "AJNR. American journal of neuroradiology"[Journal] OR "AJR. American journal of roentgenology"[Journal] OR "The British journal of radiology"[Journal] OR "Radiology"[Journal] OR "Radiographics : a review publication of the Radiological Society of North America, Inc"[Journal] OR "Neurographics (2011)"[Journal] OR "European radiology"[Journal] OR "Clinical radiology"[Journal] OR "Neuroradiology"[Journal] OR "Journal of neuroradiology = Journal de neuroradiologie"[Journal] OR "Journal of the American College of Radiology : JACR"[Journal] OR "J Neurointerv Surg"[Journal] OR "Clinical imaging"[Journal] OR "Applied radiology"[Journal] OR "european journal of radiology"[Journal] OR "investigative radiology"[Journal] OR "Academic Radiology"[Journal] OR )

AND

("2019/10/01"[Date - Publication] : "3000"[Date - Publication])

NOT

(editorial[Filter] OR letter[Filter])

PubMed=552

Embase

('COVID':ti,ab,kw OR 'COVID-19':ti,ab,kw OR 'COVID 19':ti,ab,kw OR 'COVID-2019':ti,ab,kw OR 'COVID19':ti,ab,kw OR 'SARS-CoV-2':ti,ab,kw OR 'Coronavirus':ti,ab,kw OR '2019-nCoV':ti,ab,kw OR 'nCoV-2019':ti,ab,kw OR 'coronavirus disease 2019'/exp)

AND

('neurologic':ti,ab,kw OR 'CNS':ti,ab,kw OR 'nervous system':ti,ab,kw OR 'brain':ti,ab,kw OR 'cerebr*':ti,ab,kw OR 'neurovascular':ti,ab,kw OR 'cerebrovascular':ti,ab,kw OR 'stroke':ti,ab,kw OR 'cerebral vein thrombosis':ti,ab,kw OR 'neuropath*':ti,ab,kw OR 'encephalit*':ti,ab,kw OR 'mening*':ti,ab,kw OR 'leukoencephalopath*':ti,ab,kw OR 'neurologic disease'/de OR 'Central Nervous System Diseases'/de OR 'cranial neuropathy'/de OR 'Demyelinating Diseases'/de OR 'cerebrovascular accident'/de OR 'Brain'/de OR 'nervous system'/de OR 'brain hemorrhage'/de OR 'central nervous system'/de OR 'brain'/de OR 'brain stem'/de OR 'olfactory tract'/de OR 'pituitary':ti,ab,kw OR 'intracran*':ti,ab,kw OR 'intracereb*':ti,ab,kw OR 'hemispher*':ti,ab,kw OR 'vertebrobas*':ti,ab,kw OR 'infratentorial':ti,ab,kw OR 'supratentorial':ti,ab,kw OR 'anterior circulat*':ti,ab,kw OR 'posterior circulat*':ti,ab,kw OR 'infarct*':ti,ab,kw OR 'cerebell*':ti,ab,kw OR 'cognit*':ti,ab,kw OR 'neuro*':ti,ab,kw OR 'JAMA Neurology':jt OR 'Journal of Neurology':jt OR 'Neurology':jt OR 'Journal of neurosurgery':jt OR 'Annals of neurology':jt OR 'Journal of neurointerventional surgery':jt OR 'Neurosurgery':jt OR 'The New England journal of medicine':jt OR 'Stroke':jt OR 'The Lancet. Neurology':jt OR 'Acta neuropathologica':jt OR 'Spine':jt OR 'European Journal of Neurology':jt)

AND

('Neuroimaging':ti,ab,kw OR 'Magnetic resonance imaging':ti,ab,kw OR 'MRI':ti,ab,kw OR 'computed tomograph*':ti,ab,kw OR 'CT':ti,ab,kw OR 'Imaging':ti,ab,kw OR 'CAT scan*':ti,ab,kw OR 'Magentic resonance tomography':ti,ab,kw OR 'MR Imaging':ti,ab,kw OR 'fMRI':ti,ab,kw OR 'nuclear magnetic resonance imaging'/exp OR 'x-ray computed tomography'/exp OR 'computed tomographic angiography'/de OR 'cone beam computed tomography'/de OR 'high resolution computer tomography'/de OR 'multidetector computed tomography'/de OR 'spiral computer assisted tomography'/de OR 'AJNR. American journal of neuroradiology':jt OR 'AJR. American journal of roentgenology':jt OR 'The British journal of radiology':jt OR 'Radiology':ta OR 'Radiographics':ta OR 'Neurographics (2011)':jt OR 'European radiology':jt OR 'Clinical radiology':jt OR 'Neuroradiology':jt OR 'Journal of neuroradiology. Journal de neuroradiologie':jt OR 'Journal of the American College of Radiology : JACR':jt OR 'J Neurointerv Surg':ta OR 'Clinical imaging':jt OR 'Applied radiology':jt OR 'european journal of radiology':jt OR 'investigative radiology':jt OR 'Academic Radiology':jt)

AND

([1-10-2019]/sd NOT [1-1-2022]/sd)

NOT

(‘editorial’/it OR ‘letter’/it)

Embase=634
