# Supplementary material for: Special report of the RSNA COVID-19 task force: systematic review of outcomes associated with COVID-19 neuroimaging findings in hospitalized patients
Source: Br J Radiol. 2021 Apr 29;94(1127):20210149. doi: 10.1259/bjr.20210149 (PMC8553187; doi:10.1259/bjr.20210149)
Supplement: Supplementary Material 2. [file bjr.20210149.suppl-02.docx]

**Supplementary Material: Summary of Excluded Studies with Reasons for Exclusion**

Full-text articles excluded with reasons (n = 106)

Outcome data not specific to imaging finding (n = 19)

Overlap of data reported in another paper (n = 3)

No confirmed positive COVID diagnosis reported (n = 8)

Wrong study design (i.e., review article) (n = 32)

No outcome data (n = 30)

No neuroimaging performed (n = 4)

Wrong patient population (n = 3)

Wrong setting (n = 2)

Wrong index test (n = 2)

Neuroimaging findings normal or incidental (n = 3)

| **Study** | **Reason for Exclusion** |
| --- | --- |
| Abenza-Abildúa, et al [1] | Outcome data not specific to imaging finding |
| Agarwal, et al [2] | Overlap of data reported in another paper |
| Agarwal, et al [3] | No laboratory positive COVID diagnosis reported |
| Alberti, et al [4] | Wrong study design |
| Aragao, et al [5] | No outcome data |
| Bach, et al [6] | Outcome data not specific to imaging finding |
| Baudar, et al [7] | Wrong study design |
| Behzadnia, et al [8] | No outcome data |
| Belani, et al [9] | No outcome data |
| Bersano, et al [10] | No neuroimaging performed |
| Bihlmaier, et al [11] | No laboratory positive COVID diagnosis reported |
| Boyko, et al [12] | Wrong study design |
| Castellano, et al [13] | Outcome data not specific to imaging finding |
| Chatterjee, et al [14] | No outcome data |
| Chougar, et al [15] | No outcome data |
| Chung, et al [16] | Outcome data not specific to imaging finding |
| Conklin, et al [17] | No outcome data |
| Coolen, et al [18] | Wrong patient population |
| deAlmeidaLima, et al [19] | Wrong study design |
| deOliveira, et al [20] | Wrong study design |
| Degeneffe, et al [21] | Wrong setting |
| Delorme, et al [22] | Wrong index test |
| Dhillon, et al [23] | No laboratory positive COVID diagnosis reported |
| Díaz-Pérez, et al [24] | No outcome data |
| Dinkin, et al [25] | Wrong study design |
| Dmytriw, et al [26] | Outcome data not specific to imaging finding |
| Dogra, et al [27] | Overlap of data reported in another paper |
| Egbert, et al [28] | Wrong study design |
| Eliezer, et al [29] | Wrong patient population |
| Emami, et al [30] | Neuroimaging findings normal or incidental |
| Escalard, et al [31] | Overlap of data reported in another paper |
| Esenwa, et al [32] | No outcome data |
| Faller, et al [33] | Neuroimaging findings normal or incidental |
| Fara, et al [34] | No outcome data |
| Fatehi, et al [35] | No outcome data |
| Ferrarese, et al [36] | Wrong study design |
| Fitsiori, et al [37] | No outcome data |
| Freeman, et al [38] | No outcome data |
| Frontera, et al [39] | Wrong study design |
| Garg, et al [40] | Wrong study design |
| Gulko, et al [41] | Wrong study design |
| Guven, et al [42] | Wrong study design |
| Halsey, et al [43] | Wrong index test |
| Hanif, et al [44] | Outcome data not specific to imaging finding |
| Helms, et al [45] | Outcome data not specific to imaging finding |
| Helms, et al [46] | No outcome data |
| Hossri, et al [47] | Wrong study design |
| Kadono, et al [48] | Wrong study design |
| Kandemirli, et al [49] | No outcome data |
| Katal, et al [50] | Wrong study design |
| Katz, et al [51] | Outcome data not specific to imaging finding |
| Keaney, et al [52] | No laboratory positive COVID diagnosis reported |
| Kihira, et al [53] | No outcome data |
| Kihira, et al [54] | No outcome data |
| Kihira, et al [55] | No outcome data |
| Kihira, et al [56] | No outcome data |
| Klironomos, et al [57] | Outcome data not specific to imaging finding |
| Kulick-Soperi, et al [58] | Wrong study design |
| Kwee, et al [59] | No outcome data |
| Lang, et al [60] | Outcome data not specific to imaging finding |
| Lapergue, et al [61] | No laboratory positive COVID diagnosis reported |
| Laurendon, et al [62] | Wrong study design |
| Lima, et al [63] | Wrong study design |
| Lin, et al [64] | Outcome data not specific to imaging finding |
| Lodigiani, et al [65] | Outcome data not specific to imaging finding |
| Lu, et al [66] | Wrong setting |
| Lushina, et al [67] | Wrong study design |
| Mahammedi, et al [68] | No outcome data |
| McCuddy, et al [69] | No laboratory positive COVID diagnosis reported |
| Mohammad, et al [70] | No outcome data |
| Muccioli, et al [71] | Neuroimaging findings normal or incidental |
| Nicholson, et al [72] | No outcome data |
| Novi, et al [73] | Wrong study design |
| O'Shea, et al [74] | Outcome data not specific to imaging finding |
| Padmanabhan, et al [75] | Outcome data not specific to imaging finding |
| Pan, et al [76] | Wrong study design |
| Poillon, et al [77] | No outcome data |
| Politi, et al [78] | Wrong study design |
| Poncet-Megemont, et al [79] | No neuroimaging performed |
| Pons-Escoda, et al [80] | No outcome data |
| Poyiadji, et al [81] | Wrong study design |
| Pugin, et al [82] | No laboratory positive COVID diagnosis reported |
| Pun, et al [83] | Wrong study design |
| Radmanesh, et al [84] | Outcome data not specific to imaging finding |
| Rapalino, et al [85] | No outcome data |
| Requena, et al [86] | Outcome data not specific to imaging finding |
| Sachs, et al [87] | Wrong study design |
| Schönegger, et al [88] | Wrong patient population |
| Scullen, et al [89] | No outcome data |
| Shahjouei, et al [90] | No outcome data |
| Shaw, et al [91] | Wrong study design |
| Sheth, et al [92] | No outcome data |
| Spoldi, et al [93] | No laboratory positive COVID diagnosis reported |
| Strauss, et al [94] | No outcome data |
| Studart-Neto, et al [95] | No outcome data |
| Trifan, et al [96] | Wrong study design |
| Tsivgoulis, et al [97] | Wrong study design |
| Valderrama, et al [98] | Wrong study design |
| Wijeratne, et al [99] | Wrong study design |
| Yaeger, et al [100] | Outcome data not specific to imaging finding |
| Yilmaz, et al [101] | Wrong study design |
| Yoon, et al [102] | Outcome data not specific to imaging finding |
| Zavras, et al [103] | Outcome data not specific to imaging finding |
| Zhao, et al [104] | No neuroimaging performed |
| Zhou, et al [105] | Wrong study design |
| Zuccon, et al [106] | No neuroimaging performed |

**References of excluded studies**

1. Abenza-Abildua MJ, Ramirez-Prieto MT, Moreno-Zabaleta R, Arenas-Valls N, Salvador-Maya MA, Algarra-Lucas C, et al. Neurological complications in critical patients with COVID-19. Neurologia 35 (9):621-627. doi:10.1016/j.nrl.2020.07.014

2. Agarwal S, Conway J, Nguyen V, Dogra S, Krieger P, Zagzag D, et al. Serial Imaging of Virus-Associated Necrotizing Disseminated Acute Leukoencephalopathy (VANDAL) in COVID-19. AJNR Am J Neuroradiol. doi:10.3174/ajnr.A6898

3. Agarwal S, Scher E, Rossan-Raghunath N, Marolia D, Butnar M, Torres J, et al. Acute stroke care in a New York City comprehensive stroke center during the COVID-19 pandemic. J Stroke Cerebrovasc Dis 29 (9):105068. doi:10.1016/j.jstrokecerebrovasdis.2020.105068

4. Alberti P, Beretta S, Piatti M, Karantzoulis A, Piatti ML, Santoro P, et al. Guillain-Barre syndrome related to COVID-19 infection. Neurol Neuroimmunol Neuroinflamm 7 (4). doi:10.1212/NXI.0000000000000741

5. Aragao M, Leal MC, Cartaxo Filho OQ, Fonseca TM, Valenca MM. Anosmia in COVID-19 Associated with Injury to the Olfactory Bulbs Evident on MRI. AJNR Am J Neuroradiol 41 (9):1703-1706. doi:10.3174/ajnr.A6675

6. Bach I, Surathi P, Montealegre N, Abu-Hadid O, Rubenstein S, Redko S, et al. Stroke in COVID-19: a single-centre initial experience in a hotspot of the pandemic. Stroke Vasc Neurol 5 (4):331-336. doi:10.1136/svn-2020-000525

7. Baudar C, Duprez T, Kassab A, Miller N, Rutgers MP. COVID-19 as triggering co-factor for cortical cerebral venous thrombosis? J Neuroradiol. doi:10.1016/j.neurad.2020.06.008

8. Behzadnia H, Omrani SN, Nozari-Golsefid H, Moslemi S, Alijani B, Reyhanian Z, et al. Ischemic stroke and intracerebral hemorrhage in patients with COVID-19. Romanian Journal of Neurology/ Revista Romana de Neurologie 2020;19(3):166-170.

9. Belani P, Schefflein J, Kihira S, Rigney B, Delman BN, Mahmoudi K, et al. COVID-19 Is an Independent Risk Factor for Acute Ischemic Stroke. AJNR Am J Neuroradiol 41 (8):1361-1364. doi:10.3174/ajnr.A6650

10. Bersano A, Kraemer M, Touze E, Weber R, Alamowitch S, Sibon I, et al. Stroke care during the COVID-19 pandemic: experience from three large European countries. Eur J Neurol 27 (9):1794-1800. doi:10.1111/ene.14375

11. Bihlmaier K, Coras R, Willam C, Grampp S, Jabari S, Eichhorn P, et al. Disseminated Multifocal Intracerebral Bleeding Events in Three Coronavirus Disease 2019 Patients on Extracorporeal Membrane Oxygenation As Rescue Therapy. Crit Care Explor 2 (9):e0218. doi:10.1097/CCE.0000000000000218

12. Boyko AN, Sivertseva SA, Spirin NN. Nervous system damage in COVID-19 with an emphasis on the management of patients with multiple sclerosis. Neurology, Neuropsychiatry, Psychosomatics. 2020;12(1S):44-47.

13. Castellano A, Anzalone N, Pontesilli S, Fominskiy E, Falini A. Pathological brain CT scans in severe COVID-19 ICU patients. Intensive Care Med 46 (11):2102-2104. doi:10.1007/s00134-020-06222-z

14. Chatterjee N, Mondal T, Pal, P, Mondol, K, Pal J. Covid in disguise – a series of neurological presentations. Journal of the Indian Medical Association 2020;118(5):34-36

15. Chougar L, Shor N, Weiss N, Galanaud D, Leclercq D, Mathon B, et al. Retrospective Observational Study of Brain MRI Findings in Patients with Acute SARS-CoV-2 Infection and Neurologic Manifestations. Radiology 297 (3):E313-E323. doi:10.1148/radiol.2020202422

16. Chung TW, Sridhar S, Zhang AJ, Chan KH, Li HL, Wong FK, et al. Olfactory Dysfunction in Coronavirus Disease 2019 Patients: Observational Cohort Study and Systematic Review. Open Forum Infect Dis 7 (6):ofaa199. doi:10.1093/ofid/ofaa199

17. Conklin J, Frosch MP, Mukerji S, Rapalino O, Maher M, Schaefer PW, et al. Cerebral Microvascular Injury in Severe COVID-19. medRxiv. doi:10.1101/2020.07.21.20159376

18. Coolen T, Lolli V, Sadeghi N, Rovai A, Trotta N, Taccone FS, et al. Early postmortem brain MRI findings in COVID-19 non-survivors. Neurology 95 (14):e2016-e2027. doi:10.1212/WNL.0000000000010116

19. de Almeida Lima AN, Santos Leite Pessoa M, Franco Costa Lima C, Picasso de Araujo Coimbra P, Bezerra Holanda JL. Images in Vascular Medicine: Acute peripheral artery occlusion and ischemic stroke in a patient with COVID-19. Vasc Med 25 (5):482-483. doi:10.1177/1358863X20945020

20. Oliveira RMC, Santos DH, Olivetti BC, Takahashi JT. Bilateral trochlear nerve palsy due to cerebral vasculitis related to COVID-19 infection. Arq Neuropsiquiatr 78 (6):385-386. doi:10.1590/0004-282X20200052

21. Degeneffe A, Bruneau M, Spitaels J, Gilis N, De Witte O, Lubansu A. Acute Hemorrhage After Intracerebral Biopsy in COVID-19 Patients: Report of 3 Cases. World Neurosurg 141:157-161. doi:10.1016/j.wneu.2020.06.016

22. Delorme C, Paccoud O, Kas A, Hesters A, Bombois S, Shambrook P, et al. COVID-19-related encephalopathy: a case series with brain FDG-positron-emission tomography/computed tomography findings. Eur J Neurol 27 (12):2651-2657. doi:10.1111/ene.14478

23. Dhillon PS, Chattopadhyay A, Dineen RA, Lenthall R. Hemorrhagic Neurologic Manifestations in COVID-19: An Isolated or Multifactorial Cause? AJNR Am J Neuroradiol 41 (11):E89-E90. doi:10.3174/ajnr.A6795

24. Diaz-Perez C, Ramos C, Lopez-Cruz A, Munoz Olmedo J, Lazaro Gonzalez J, De Vega-Rios E, et al. Acutely altered mental status as the main clinical presentation of multiple strokes in critically ill patients with COVID-19. Neurol Sci 41 (10):2681-2684. doi:10.1007/s10072-020-04679-w

25. Dinkin M, Gao V, Kahan J, Bobker S, Simonetto M, Wechsler P, et al. COVID-19 presenting with ophthalmoparesis from cranial nerve palsy. Neurology 95 (5):221-223. doi:10.1212/WNL.0000000000009700

26. Dmytriw AA, Phan K, Schirmer C, Settecase F, Heran MKS, Efendizade A, et al. Ischaemic stroke associated with COVID-19 and racial outcome disparity in North America. J Neurol Neurosurg Psychiatry 91 (12):1362-1364. doi:10.1136/jnnp-2020-324653

27. Dogra S, Jain R, Cao M, Bilaloglu S, Zagzag D, Hochman S, et al. Hemorrhagic stroke and anticoagulation in COVID-19. J Stroke Cerebrovasc Dis 29 (8):104984. doi:10.1016/j.jstrokecerebrovasdis.2020.104984

28. Egbert AR, Cankurtaran S, Karpiak S. Brain abnormalities in COVID-19 acute/subacute phase: A rapid systematic review. Brain Behav Immun 89:543-554. doi:10.1016/j.bbi.2020.07.014

29. Eliezer M, Hamel AL, Houdart E, Herman P, Housset J, Jourdaine C, et al. Loss of smell in patients with COVID-19: MRI data reveal a transient edema of the olfactory clefts. Neurology 95 (23):e3145-e3152. doi:10.1212/WNL.0000000000010806

30. Emami A, Fadakar N, Akbari A, Lotfi M, Farazdaghi M, Javanmardi F, et al. Seizure in patients with COVID-19. Neurol Sci 41 (11):3057-3061. doi:10.1007/s10072-020-04731-9

31. Escalard S, Maier B, Redjem H, Delvoye F, Hebert S, Smajda S, et al. Treatment of Acute Ischemic Stroke due to Large Vessel Occlusion With COVID-19: Experience From Paris. Stroke 51 (8):2540-2543. doi:10.1161/STROKEAHA.120.030574

32. Esenwa C, Cheng NT, Lipsitz E, Hsu K, Zampolin R, Gersten A, et al. COVID-19-Associated Carotid Atherothrombosis and Stroke. AJNR Am J Neuroradiol 41 (11):1993-1995. doi:10.3174/ajnr.A6752

33. Faller E, Lapthorne S, Barry R, Shamile F, Salleh F, Doyle D, et al. The Presentation and Diagnosis of the First Known Community-Transmitted Case of SARS-CoV-2 in the Republic of Ireland. Ir Med J 113 (5):78

34. Fara MG, Stein LK, Skliut M, Morgello S, Fifi JT, Dhamoon MS. Macrothrombosis and stroke in patients with mild Covid-19 infection. J Thromb Haemost 18 (8):2031-2033. doi:10.1111/jth.14938

35. Fatehi P, Hesam-Shariati N, Abouzaripour M, Fathi F, Hesam Shariati MB. Acute Ischemic and Hemorrhagic Stroke and COVID-19: Case Series. SN Compr Clin Med:1-6. doi:10.1007/s42399-020-00559-8

36. Ferrarese C, Silani V, Priori A, Galimberti S, Agostoni E, Monaco S, et al. An Italian multicenter retrospective-prospective observational study on neurological manifestations of COVID-19 (NEUROCOVID). Neurol Sci 41 (6):1355-1359. doi:10.1007/s10072-020-04450-1

37. Fitsiori A, Pugin D, Thieffry C, Lalive P, Vargas MI. COVID-19 is Associated with an Unusual Pattern of Brain Microbleeds in Critically Ill Patients. J Neuroimaging 30 (5):593-597. doi:10.1111/jon.12755

38. Freeman CW, Masur J, Hassankhani A, Wolf RL, Levine JM, Mohan S. COVID-19-Related Disseminated Leukoencephalopathy (CRDL): A Retrospective Study of Findings on Brain MRI. AJR Am J Roentgenol. doi:10.2214/AJR.20.24364

39. Frontera J, Mainali S, Fink EL, Robertson CL, Schober M, Ziai W, et al. Global Consortium Study of Neurological Dysfunction in COVID-19 (GCS-NeuroCOVID): Study Design and Rationale. Neurocrit Care 33 (1):25-34. doi:10.1007/s12028-020-00995-3

40. Garg RK, Paliwal VK, Gupta A. Encephalopathy in patients with COVID-19: A review. J Med Virol. doi:10.1002/jmv.26207

41. Gulko E, Oleksk ML, Gomes W, Ali S, Mehta H, Overby P, et al. MRI Brain Findings in 126 Patients with COVID-19: Initial Observations from a Descriptive Literature Review. AJNR Am J Neuroradiol 41 (12):2199-2203. doi:10.3174/ajnr.A6805

42. Guven F, Ogul H, Turgut A, Tezcan A, Kantarci M. Leptomeningeal involvement in a patient with systemic lupus erythematosus infected by COVID-19. Joint Bone Spine 87 (5):495. doi:10.1016/j.jbspin.2020.06.002

43. Halsey R, Priftakis D, Mackenzie S, Wan S, Davis LM, Lilburn D, et al. COVID-19 in the act: incidental 18F-FDG PET/CT findings in asymptomatic patients and those with symptoms not primarily correlated with COVID-19 during the United Kingdom coronavirus lockdown. Eur J Nucl Med Mol Imaging. doi:10.1007/s00259-020-04972-y

44. Hanif A, Khan S, Mantri N, Hanif S, Saleh M, Alla Y, et al. Thrombotic complications and anticoagulation in COVID-19 pneumonia: a New York City hospital experience. Ann Hematol 99 (10):2323-2328. doi:10.1007/s00277-020-04216-x

45. Helms J, Kremer S, Merdji H, Schenck M, Severac F, Clere-Jehl R, et al. Delirium and encephalopathy in severe COVID-19: a cohort analysis of ICU patients. Crit Care 24 (1):491. doi:10.1186/s13054-020-03200-1

46. Helms J, Tacquard C, Severac F, Leonard-Lorant I, Ohana M, Delabranche X, et al. High risk of thrombosis in patients with severe SARS-CoV-2 infection: a multicenter prospective cohort study. Intensive Care Med 46 (6):1089-1098. doi:10.1007/s00134-020-06062-x

47. Hossri S, Shadi M, Hamarsha Z, Schneider R, El-Sayegh D. Clinically significant anticardiolipin antibodies associated with COVID-19. J Crit Care 59:32-34. doi:10.1016/j.jcrc.2020.05.017

48. Kadono Y, Nakamura Y, Ogawa Y, Yamamoto S, Kajikawa R, Nakajima Y, et al. A case of COVID-19 infection presenting with a seizure following severe brain edema. Seizure 80:53-55. doi:10.1016/j.seizure.2020.06.015

49. Kandemirli SG, Dogan L, Sarikaya ZT, Kara S, Akinci C, Kaya D, et al. Brain MRI Findings in Patients in the Intensive Care Unit with COVID-19 Infection. Radiology 297 (1):E232-E235. doi:10.1148/radiol.2020201697

50. Katal S, Balakrishnan S, Gholamrezanezhad A. Neuroimaging and neurologic findings in COVID-19 and other coronavirus infections: A systematic review in 116 patients. J Neuroradiol. doi:10.1016/j.neurad.2020.06.007

51. Katz JM, Libman RB, Wang JJ, Filippi CG, Sanelli P, Zlochower A, et al. COVID-19 Severity and Stroke: Correlation of Imaging and Laboratory Markers. AJNR Am J Neuroradiol. doi:10.3174/ajnr.A6920

52. Keaney K, Mumtaz T. Cerebral venous thrombosis in patients with severe COVID-19 infection in intensive care. Br J Hosp Med (Lond) 81 (9):1-4. doi:10.12968/hmed.2020.0327

53. Kihira S, Delman BN, Belani P, Stein L, Aggarwal A, Rigney B, et al. Imaging Features of Acute Encephalopathy in Patients with COVID-19: A Case Series. AJNR Am J Neuroradiol 41 (10):1804-1808. doi:10.3174/ajnr.A6715

54. Kihira S, Schefflein J, Chung M, Mahmoudi K, Rigney B, Delman BN, et al. Incidental COVID-19 related lung apical findings on stroke CTA during the COVID-19 pandemic. J Neurointerv Surg 12 (7):669-672. doi:10.1136/neurintsurg-2020-016188

55. Kihira S, Schefflein J, Mahmoudi K, Rigney B, B ND, Mocco J, Doshi A, et al. Association of Coronavirus Disease (COVID-19) With Large Vessel Occlusion Strokes: A Case-Control Study. AJR Am J Roentgenol 216 (1):150-156. doi:10.2214/AJR.20.23847

56. Kihira S, Schefflein J, Pawha P, Rigney B, Delman BN, Xuan D, et al. Neurovascular complications that can be seen in COVID-19 patients. Clin Imaging 69:280-284. doi:10.1016/j.clinimag.2020.09.011

57. Klironomos S, Tzortzakakis A, Kits A, Ohberg C, Kollia E, Ahoromazdae A, et al. Nervous System Involvement in Coronavirus Disease 2019: Results from a Retrospective Consecutive Neuroimaging Cohort. Radiology 297 (3):E324-E334. doi:10.1148/radiol.2020202791

58. Kulick-Soper CV, McKee JL, Wolf RL, Mohan S, Stein JM, Masur JH, et al. Pearls & Oy-sters: Bilateral globus pallidus lesions in a patient with COVID-19. Neurology 95 (10):454-457. doi:10.1212/WNL.0000000000010157

59. Kwee RM, Krdzalic J, Fasen B, de Jaegere TMH, Group C-CIS-ENS. CT Scanning in Suspected Stroke or Head Trauma: Is it Worth Going the Extra Mile and Including the Chest to Screen for COVID-19 Infection? AJNR Am J Neuroradiol 41 (7):1165-1169. doi:10.3174/ajnr.A6607

60. Lang M, Li MD, Buch K, Yoon BC, Applewhite BP, Leslie-Mazwi TM, et al. Risk of Acute Cerebrovascular Events in Patients with COVID-19 Infection. AJNR Am J Neuroradiol 41 (11):E92-E93. doi:10.3174/ajnr.A6796

61. Lapergue B, Lyoubi A, Meseguer E, Avram I, Denier C, Venditti L, et al. Large vessel stroke in six patients following SARS-CoV-2 infection: a retrospective case study series of acute thrombotic complications on stable underlying atherosclerotic disease. Eur J Neurol 27 (11):2308-2311. doi:10.1111/ene.14466

62. Laurendon T, Radulesco T, Mugnier J, Gerault M, Chagnaud C, El Ahmadi AA, et al. Bilateral transient olfactory bulb edema during COVID-19-related anosmia. Neurology 95 (5):224-225. doi:10.1212/WNL.0000000000009850

63. Lima CFC, Holanda JLB, Pessoa MSL, Coimbra PPA. Acute ischemic stroke in a patient with COVID-19. Arq Neuropsiquiatr 78 (7):454-455. doi:10.1590/0004-282x20200057

64. Lin E, Lantos JE, Strauss SB, Phillips CD, Campion TR, Jr., Navi BB, et al. Brain Imaging of Patients with COVID-19: Findings at an Academic Institution during the Height of the Outbreak in New York City. AJNR Am J Neuroradiol 41 (11):2001-2008. doi:10.3174/ajnr.A6793

65. Lodigiani C, Iapichino G, Carenzo L, Cecconi M, Ferrazzi P, Sebastian T, et al. Venous and arterial thromboembolic complications in COVID-19 patients admitted to an academic hospital in Milan, Italy. Thromb Res 191:9-14. doi:10.1016/j.thromres.2020.04.024

66. Lu Y, Li X, Geng D, Mei N, Wu PY, Huang CC, et al. Cerebral Micro-Structural Changes in COVID-19 Patients - An MRI-based 3-month Follow-up Study. EClinicalMedicine 25:100484. doi:10.1016/j.eclinm.2020.100484

67. Lushina N, Kuo JS, Shaikh HA. Pulmonary, Cerebral, and Renal Thromboembolic Disease in a Patient with COVID-19. Radiology 296 (3):E181-E183. doi:10.1148/radiol.2020201623

68. Mahammedi A, Saba L, Vagal A, Leali M, Rossi A, Gaskill M, et al. Imaging of Neurologic Disease in Hospitalized Patients with COVID-19: An Italian Multicenter Retrospective Observational Study. Radiology 297 (2):E270-E273. doi:10.1148/radiol.2020201933

69. McCuddy M, Kelkar P, Zhao Y, Wicklund D. Acute Demyelinating Encephalomyelitis (ADEM) in COVID-19 Infection: A Case Series. Neurol India 68 (5):1192-1195. doi:10.4103/0028-3886.299174

70. Mohammad LM, Botros JA, Chohan MO. Necessity of brain imaging in COVID-19 infected patients presenting with acute neurological deficits. Interdiscip Neurosurg 22:100883. doi:10.1016/j.inat.2020.100883

71. Muccioli L, Pensato U, Bernabe G, Ferri L, Tappata M, Volpi L, et al. Intravenous immunoglobulin therapy in COVID-19-related encephalopathy. J Neurol. doi:10.1007/s00415-020-10248-0

72. Nicholson P, Alshafai L, Krings T. Neuroimaging Findings in Patients with COVID-19. AJNR Am J Neuroradiol 41 (8):1380-1383. doi:10.3174/ajnr.A6630

73. Novi G, Rossi T, Pedemonte E, Saitta L, Rolla C, Roccatagliata L, et al. Acute disseminated encephalomyelitis after SARS-CoV-2 infection. Neurol Neuroimmunol Neuroinflamm 7 (5). doi:10.1212/NXI.0000000000000797

74. O'Shea A, Parakh A, Hedgire S, Lee SI. Multisystem assessment of the imaging manifestations of coagulopathy in hospitalized patients with COVID-19. AJR Am J Roentgenol. doi:10.2214/AJR.20.24132

75. Padmanabhan N, Natarajan I, Gunston R, Raseta M, Roffe C. Impact of COVID-19 on stroke admissions, treatments, and outcomes at a comprehensive stroke centre in the United Kingdom. Neurol Sci. doi:10.1007/s10072-020-04775-x

76. Pan S, Chen WC, Baal JD, Sugrue LP. Neuroradiological Features of Mild and Severe SARS-CoV-2 Infection. Acad Radiol 27 (11):1507-1514. doi:10.1016/j.acra.2020.08.026

77. Poillon G, Obadia M, Perrin M, Savatovsky J, Lecler A. Cerebral venous thrombosis associated with COVID-19 infection: Causality or coincidence? J Neuroradiol. doi:10.1016/j.neurad.2020.05.003

78. Politi LS, Salsano E, Grimaldi M. Magnetic Resonance Imaging Alteration of the Brain in a Patient With Coronavirus Disease 2019 (COVID-19) and Anosmia. JAMA Neurol 77 (8):1028-1029. doi:10.1001/jamaneurol.2020.2125

79. Poncet-Megemont L, Paris P, Tronchere A, Salazard JP, Pereira B, Dallel R, et al. High Prevalence of Headaches During Covid-19 Infection: A Retrospective Cohort Study. Headache 60 (10):2578-2582. doi:10.1111/head.13923

80. Pons-Escoda A, Naval-Baudin P, Majos C, Camins A, Cardona P, Cos M, et al. Neurologic Involvement in COVID-19: Cause or Coincidence? A Neuroimaging Perspective. AJNR Am J Neuroradiol 41 (8):1365-1369. doi:10.3174/ajnr.A6627

81. Poyiadji N, Shahin G, Noujaim D, Stone M, Patel S, Griffith B. COVID-19-associated Acute Hemorrhagic Necrotizing Encephalopathy: Imaging Features. Radiology 296 (2):E119-E120. doi:10.1148/radiol.2020201187

82. Pugin D, Vargas MI, Thieffry C, Schibler M, Grosgurin O, Pugin J, et al. COVID-19-related encephalopathy responsive to high-dose glucocorticoids. Neurology 95 (12):543-546. doi:10.1212/WNL.0000000000010354

83. Pun M, Haggerty-Skeans J, Pratt D, Fudym Y, Al-Holou WN, Camelo-Piragua S, et al. H3K27M-mutant diffuse midline glioma with extensive intratumoral microthrombi in a young adult with COVID-19-associated coagulopathy. Acta Neuropathol 140 (2):227-229. doi:10.1007/s00401-020-02184-0

84. Radmanesh A, Raz E, Zan E, Derman A, Kaminetzky M. Brain Imaging Use and Findings in COVID-19: A Single Academic Center Experience in the Epicenter of Disease in the United States. AJNR Am J Neuroradiol 41 (7):1179-1183. doi:10.3174/ajnr.A6610

85. Rapalino O, Weerasekera A, Moum SJ, Eikermann-Haerter K, Edlow BL, Fischer D, et al. Brain MR Spectroscopic Findings in 3 Consecutive Patients with COVID-19: Preliminary Observations. AJNR Am J Neuroradiol. doi:10.3174/ajnr.A6877

86. Requena M, Olive-Gadea M, Muchada M, Garcia-Tornel A, Deck M, Juega J, et al. COVID-19 and Stroke: Incidence and Etiological Description in a High-Volume Center. J Stroke Cerebrovasc Dis 29 (11):105225. doi:10.1016/j.jstrokecerebrovasdis.2020.105225

87. Sachs JR, Gibbs KW, Swor DE, Sweeney AP, Williams DW, Burdette JH, et al. COVID-19-associated Leukoencephalopathy. Radiology 296 (3):E184-E185. doi:10.1148/radiol.2020201753

88. Schonegger CM, Gietl S, Heinzle B, Freudenschuss K, Walder G. Smell and Taste Disorders in COVID-19 Patients: Objective Testing and Magnetic Resonance Imaging in Five Cases. SN Compr Clin Med:1-5. doi:10.1007/s42399-020-00606-4

89. Scullen T, Keen J, Mathkour M, Dumont AS, Kahn L. Coronavirus 2019 (COVID-19)-Associated Encephalopathies and Cerebrovascular Disease: The New Orleans Experience. World Neurosurg 141:e437-e446. doi:10.1016/j.wneu.2020.05.192

90. Shahjouei S, Naderi S, Li J, Khan A, Chaudhary D, Farahmand G, et al. Risk of stroke in hospitalized SARS-CoV-2 infected patients: A multinational study. EBioMedicine 59:102939. doi:10.1016/j.ebiom.2020.102939

91. Shaw VC, Chander G, Puttanna A. Neuromyelitis optica spectrum disorder secondary to COVID-19. Br J Hosp Med (Lond) 81 (9):1-3. doi:10.12968/hmed.2020.0401

92. Sheth KN, Mazurek MH, Yuen MM, Cahn BA, Shah JT, Ward A, et al. Assessment of Brain Injury Using Portable, Low-Field Magnetic Resonance Imaging at the Bedside of Critically Ill Patients. JAMA Neurol. doi:10.1001/jamaneurol.2020.3263

93. Spoldi C, Castellani L, Pipolo C, Maccari A, Lozza P, Scotti A, et al. Isolated olfactory cleft involvement in SARS-CoV-2 infection: prevalence and clinical correlates. Eur Arch Otorhinolaryngol. doi:10.1007/s00405-020-06165-7

94. Strauss SB, Lantos JE, Heier LA, Shatzkes DR, Phillips CD. Olfactory Bulb Signal Abnormality in Patients with COVID-19 Who Present with Neurologic Symptoms. AJNR Am J Neuroradiol 41 (10):1882-1887. doi:10.3174/ajnr.A6751

95. Studart-Neto A, Guedes BF, Tuma RLE, Camelo Filho AE, Kubota GT, Iepsen BD, et al. Neurological consultations and diagnoses in a large, dedicated COVID-19 university hospital. Arq Neuropsiquiatr 78 (8):494-500. doi:10.1590/0004-282X20200089

96. Trifan G, Hillmann M, Testai FD. Acute Stroke as the Presenting Symptom of SARS-CoV-2 Infection in a Young Patient with Cerebral Autosomal Dominant Arteriopathy with Subcortical Infarcts and Leukoencephalopathy. J Stroke Cerebrovasc Dis 29 (10):105167. doi:10.1016/j.jstrokecerebrovasdis.2020.105167

97. Tsivgoulis G, Fragkou PC, Lachanis S, Palaiodimou L, Lambadiari V, Papathanasiou M, et al. Olfactory bulb and mucosa abnormalities in persistent COVID-19-induced anosmia: a magnetic resonance imaging study. Eur J Neurol 28 (1):e6-e8. doi:10.1111/ene.14537

98. Valderrama EV, Humbert K, Lord A, Frontera J, Yaghi S. Severe Acute Respiratory Syndrome Coronavirus 2 Infection and Ischemic Stroke. Stroke 51 (7):e124-e127. doi:10.1161/STROKEAHA.120.030153

99. Wijeratne T, Sales C, Karimi L, Crewther SG. Acute Ischemic Stroke in COVID-19: A Case-Based Systematic Review. Front Neurol 11:1031. doi:10.3389/fneur.2020.01031

100. Yaeger KA, Fifi JT, Lara-Reyna J, Rossitto C, Ladner T, Yim B, et al. Initial Stroke Thrombectomy Experience in New York City during the COVID-19 Pandemic. AJNR Am J Neuroradiol 41 (8):1357-1360. doi:10.3174/ajnr.A6652

101. Yilmaz U, Lepper PM, Reith W. COVID-19: neurological manifestations : What we know so far. Radiologe 60 (10):916-918. doi:10.1007/s00117-020-00748-5

102. Yoon BC, Buch K, Lang M, Applewhite BP, Li MD, Mehan WA, et al. Clinical and Neuroimaging Correlation in Patients with COVID-19. AJNR Am J Neuroradiol 41 (10):1791-1796. doi:10.3174/ajnr.A6717

103. Zavras PD, Kabarriti R, Mehta V, Goel S, Billett HH. Clinical Thrombosis Rate was not Increased in a Cohort of Cancer Patients with COVID-19. medRxiv. doi:10.1101/2020.09.15.20195263

104. Zhao M, Wang M, Zhang J, Gu J, Zhang P, Xu Y, et al. Comparison of clinical characteristics and outcomes of patients with coronavirus disease 2019 at different ages. Aging (Albany NY) 12 (11):10070-10086. doi:10.18632/aging.103298

105. Zhou S, Jones-Lopez EC, Soneji DJ, Azevedo CJ, Patel VR. Myelin Oligodendrocyte Glycoprotein Antibody-Associated Optic Neuritis and Myelitis in COVID-19. J Neuroophthalmol 40 (3):398-402. doi:10.1097/WNO.0000000000001049

106. Zuccon W, Comassi P, Adriani L, Bergamaschini G, Bertin E, Borromeo R, et al. Intensive care for seriously ill patients affected by novel coronavirus sars - CoV - 2: Experience of the Crema Hospital, Italy. Am J Emerg Med. doi:10.1016/j.ajem.2020.08.005
